# Supplementary material for: Antifungal tolerance is a subpopulation effect distinct from resistance and is associated with persistent candidemia
Source: Nat Commun. 2018 Jun 25;9:2470. doi: 10.1038/s41467-018-04926-x (PMC6018213; doi:10.1038/s41467-018-04926-x)
Supplement: Supplementary file 1 — Supplementary Information [file 41467_2018_4926_MOESM1_ESM.pdf]

# Antifungal tolerance is a subpopulation effect distinct from resistance and is associated with persistent candidemia

Rosenberg and Ene, et al.

## Supplementary information

**Supplementary Note 1. Cell viability within the zone of inhibition of disk diffusion assays.** Replica-plating of cells from disk diffusion assays (DDAs) to drug-free medium produced an even lawn of growing cells (Fig. 2h and Supplementary Fig. 7h), revealing that the majority of cells were viable even when exposed to high drug concentrations. Viability was also measured by staining cells with the vital dye Propidium iodide (PI), which selectively permeates dead cells[1]. These assays similarly indicated that the proportion of dead cells taken from inside or outside the zone of inhibition were comparable, with only 1-2% showing the bright PI staining indicative of dead cells. Importantly, this value was independent of the relative FoG levels of tested strains (Supplementary Table 1).

**Supplementary Note 2. Effect of *IRO1* on tolerance in SC5314-derived strains.** We first measured FoG across a set of SC5314 derived laboratory strains (Supplementary Fig. 8a). FoG levels differed by 40-50% between these strains (e.g., CAI-4 and SN76) relative to SC5314 and this difference was largely attributable to *IRO1,* which was deleted in some of the isolates (Supplementary Fig. 8b and Supplementary Table 2). *IRO1* encodes a transcription factor involved in iron utilization[2]. Tolerance might require iron availability as (1) Erg11, the cytochrome p450 target of FLC requires a heme group; (2) expression of *HSP90* and *CRZ1* via calcineurin signalling[3] is regulated by iron deprivation; and (3) *RIM101,* a transcription factor that responds to pH and iron levels[4], also regulates *HSP90* expression[5].

**Supplementary Note 3. Effect of signaling pathways on tolerance and resistance.** Stress response strategies are <u>indirect</u> mechanisms that may enable survival of cells despite the continued presence of the drug and the interaction with its target. We

examined the role of *C. albicans* stress response pathways on tolerance using DDAs. FoG measures properties previously termed ‘Hsp90-dependent response’, which are distinct from *bona fide* resistance. Hsp90 and its client protein calcineurin are known to affect fungal drug responses, including the cidality of azoles[6-13]. As expected, FoG was cleared in mutants lacking either the calcineurin subunit Cnb1[14], or Crz1, the transcription factor downstream of calcineurin[15]. Importantly, neither of these mutants affected RAD/MIC levels (Fig. 4d and Supplementary Table 3). Deletion of other calcineurin regulators, including *RCN1*[16] or *RCN2*[17], had little effect on either RAD or FoG levels (Fig. 4d), indicating that they likely have redundant functions[16].

Tolerance also requires the PKC signalling cascade (Fig. 4a,b, see results with staurosporine), therefore we tested the effects of Mkc1[18], the terminal MAP-kinase of the Pkc pathway, in DDAs. Deletion of *MKC1* dramatically reduced FoG along with a minor increase in RAD, suggesting that Mkc1 primarily affects tolerance (Fig. 4d). This is consistent with previous reports indicating that Mkc1 contributes to Hsp90-dependent ‘resistance’[19]. Vps21, a Rab GTPase that regulates endosomal trafficking through the late endosome was also reported to affect tolerance and not MIC[20,21]**.** Accordingly[21], loss of *VPS21* increased FoG levels and did not affect RAD (Fig. 4d). We also tested genes that affect major mechanisms of azole resistance: Upc2 is a transcription factor that regulates the expression of ergosterol biosynthetic genes and drug efflux pumps[22-25]. *upc2*Δ mutants displayed increased RAD and greatly reduced FoG (Fig. 4e). By contrast, loss of Tac1 and Mrr1, transcription factors that upregulate ABC- and MFS- transporter expression, had no effect on either RAD or FoG (Fig. 4e). This is likely because multiple efflux pumps are under complex, yet redundant transcriptional control and are only slightly induced by exposure to fluconazole[24,26,27].

Upc2 affects resistance by regulating the expression of *ERG* genes (and its own expression)[28] in response to reduced ergosterol[28]. But how does Upc2 affect FoG? One possibility is that Upc2 may regulate multiple independent pathways, some of which influence susceptibility and others that affect tolerance. Indeed, deletion of *UPC2* directly affects the *ERG* genes involved in sterol biosynthesis as well as genes important for iron homeostasis[22] and also affects the process of NLRP3-mediated pyroptosis in macrophages[29]. Alternatively, Upc2 may also affect the degree of cellular stress in response to FLC and thus may alter the requirement for the stress response mechanisms that drive tolerance. This is consistent with the general observation that compounds and mutants that dramatically reduce the MIC usually also clear FoG.

Taken together, FoG is modulated by genetic pathways previously reported to control Hsp90-dependent 'resistance' and/or 'tolerance'. Moreover, multiple factors specifically impact FoG without affecting conventional susceptibility/resistance levels as measured by RAD, underlining the distinction between tolerance and resistance.

**Supplementary Note 4. Use of doxycycline when evaluating drug responses.** Interestingly, doxycycline reduces tolerance by chelating limited iron stores in the medium[10], and iron deprivation regulates the expression of *HSP90* and *CRZ1* via calcineurin signalling[3]. The effect of doxycycline on iron availability is important to consider when studying azole responses using doxycycline to activate or repress the Tet promoter in engineered strains[30,31].

## Supplementary Figures

### Supplementary Figure 1

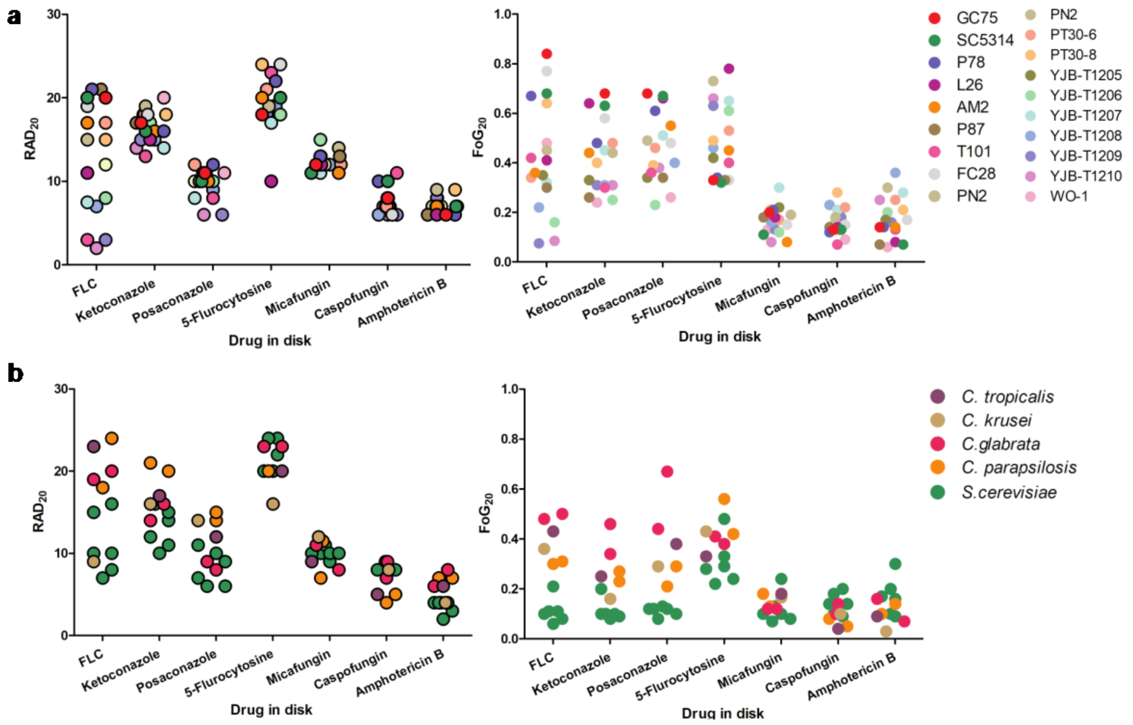


**Supplementary Figure 1.** Tolerance is detected for different drugs and fungal species. (a) DDAs measure RAD and FoG levels for a set of 19 *C. albicans* isolates in response to diverse antifungal drugs including FLC (25 µg), ketoconazole (10 µg), posaconazole (10 µg), 5-fluorocytosine (25 µg), micafungin (5 µg), caspofungin (5 µg), or amphotericin B (50 µg). (b) DDAs performed with all the above mentioned antifungal disks to detect RAD and FoG levels for different pathogenic yeast species including *C. tropicalis*, *C. krusei*, *C. glabrata*, *C. parapsilosis* and *S. cerevisiae*. Note that FoG levels in azoles were particularly low for *S. cerevisiae.* For all panels, $n \geq 2$.

## Supplementary Figure 2

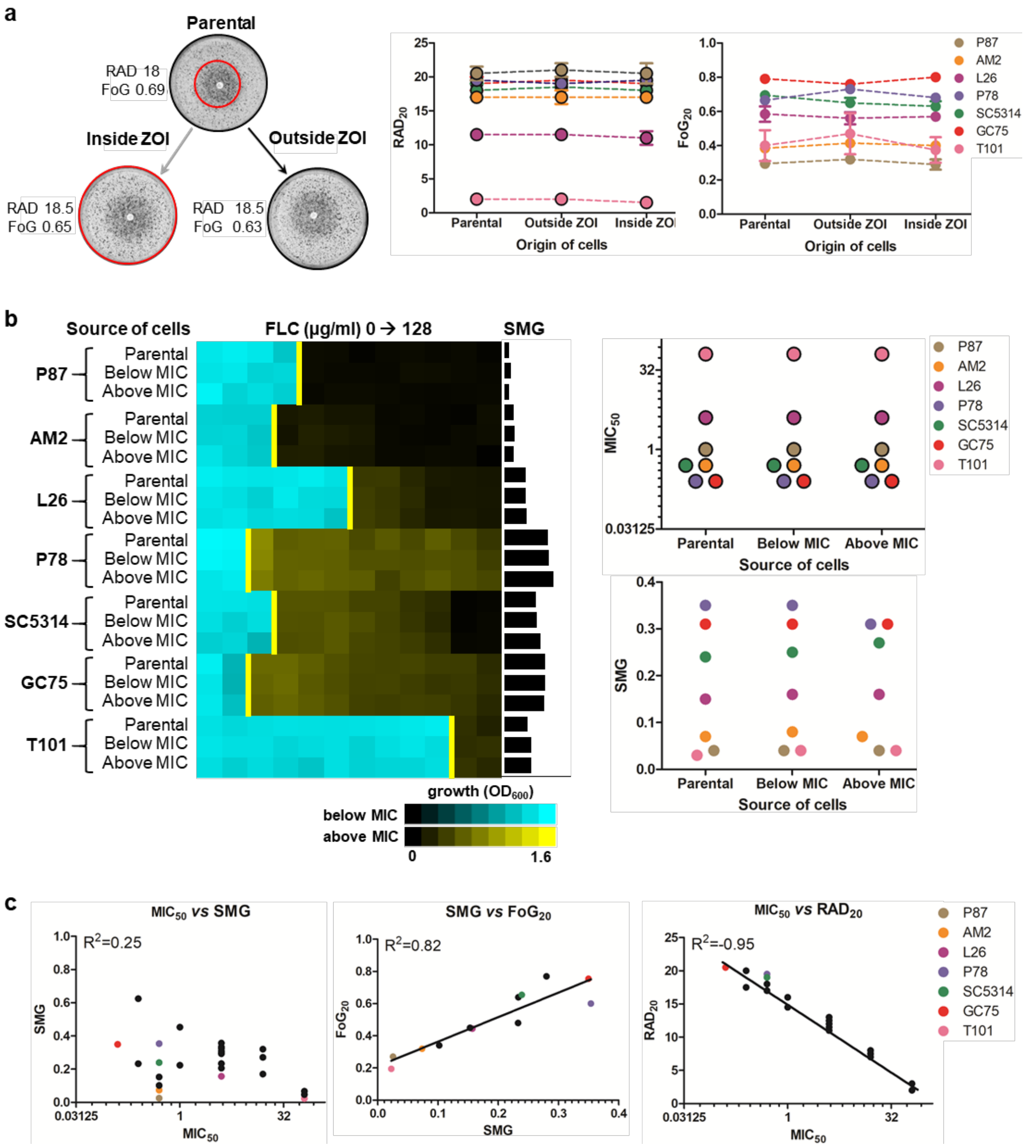


**Supplementary Figure 2.** Measuring heritability and drug responses of *C. albicans* isolates using DDAs and broth microdilution assays (BMDAs). (a) $RAD_{20}$ and $FoG_{20}$ for a parental strain and progeny colonies picked from inside and outside the zone of inhibition. Left panel, DDAs for strain SC5314, and corresponding RAD (middle) and FoG (right) levels for diverse *C. albicans* isolates. RAD (Paired t-test, n pairs=7, *P* =

0.0656), FoG (Paired t-test, n pairs=7, *P* - 0.0138). Note that both RAD and FoG are indistinguishable between the two plates for all strains (right panels); (b) Comparison of MIC and SMG for parental cells and cells growing at drug concentrations below (cyan heatmaps) or above (yellow heatmaps) $MIC_{50}$ levels (yellow lines). MIC (Paired t-test, n pairs=7, $P = 0.2308$), SMG (Paired t-test, n pairs=7, $P = 0.0603$). Again, MIC and SMG are indistinguishable between the wells above and below $MIC_{50}$ for all strains (right panels). (c) Correlation analyses for MIC and SMG (n=28, $P = 0.0064$), SMG and FoG (n=12, $P < 0.0001$), MIC and RAD (n=28, $P < 0.0001$) for the series of isolates analysed in Fig. 1. For all panels, $n \geq 2$ and error bars denote standard deviations.

## Supplementary Figure 3

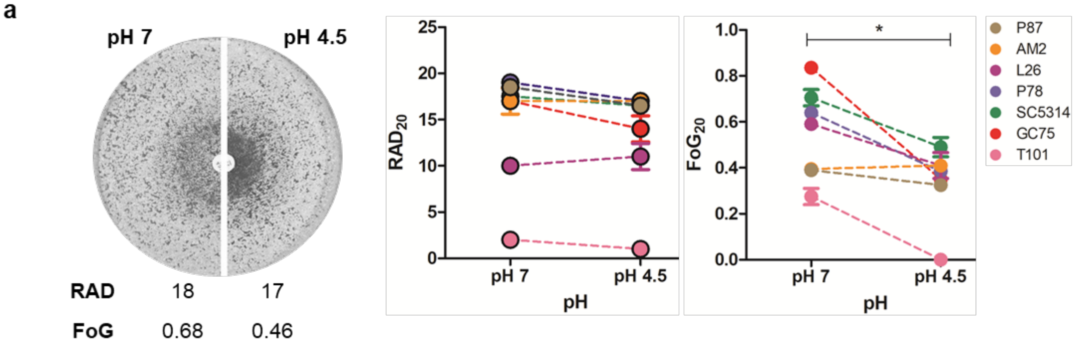


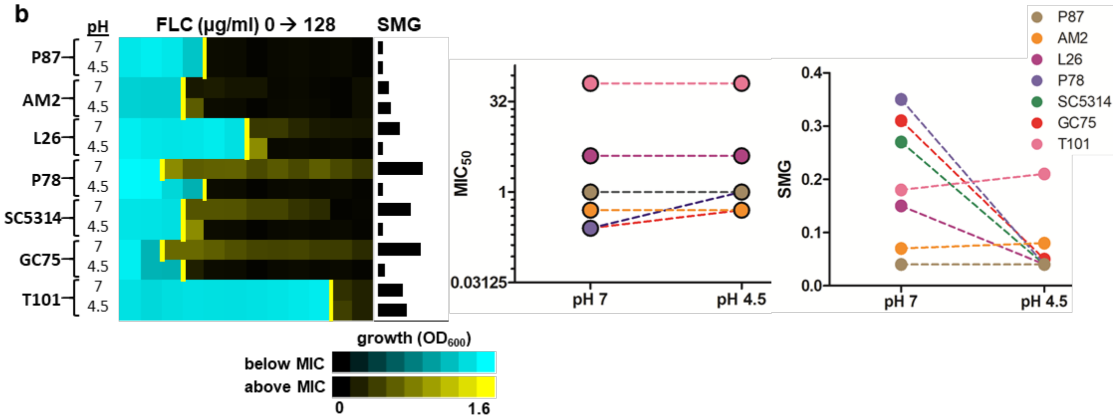


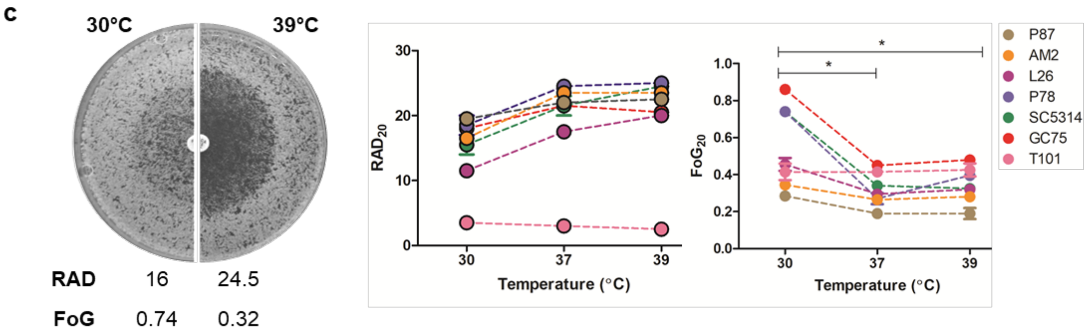

**Supplementary Figure 3, continued**

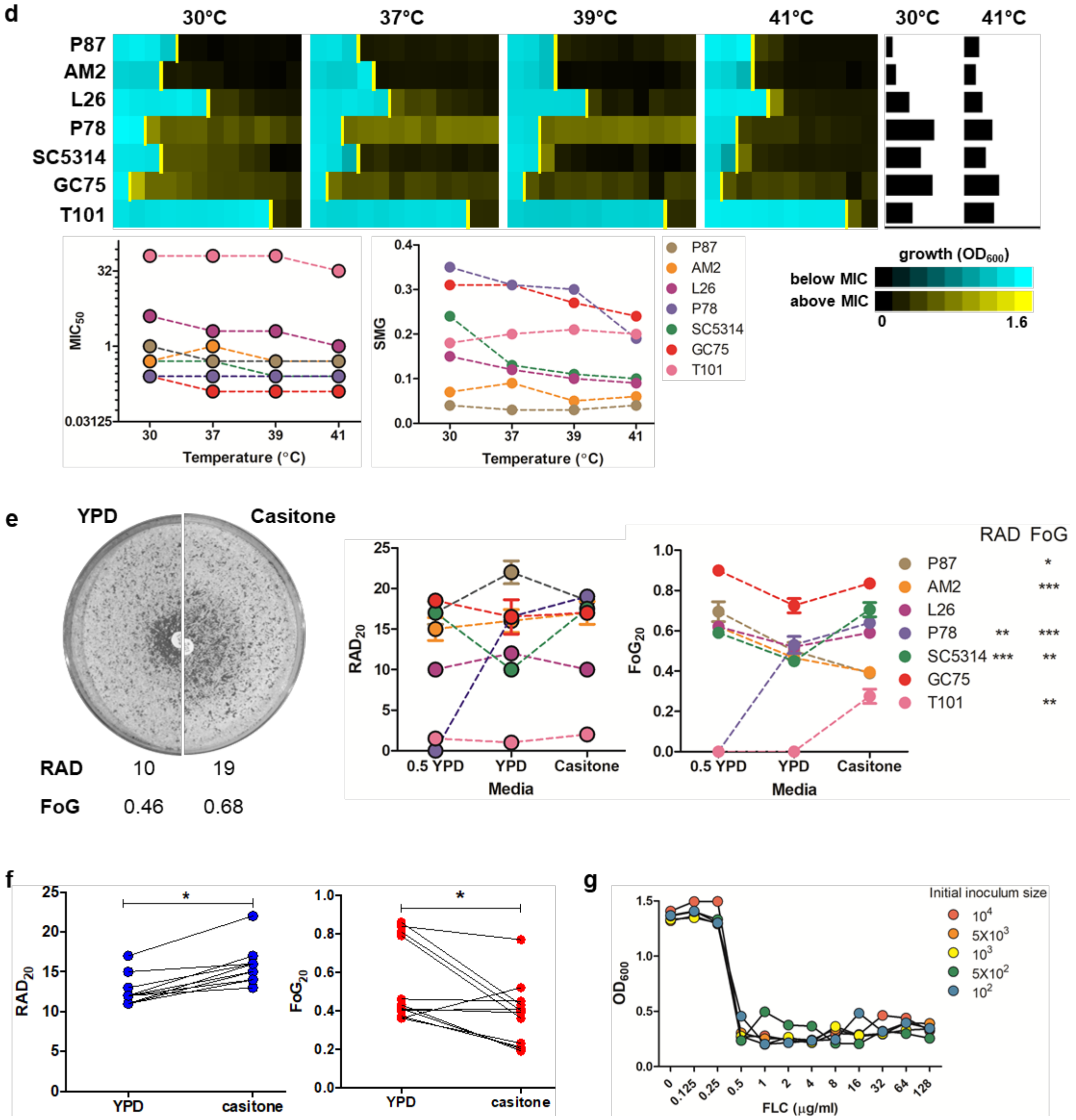


**Supplementary Figure 3.** Impact of growth conditions on FoG and SMG levels. (a,b) DDAs (a) and BMDAs (b) measuring the effect of pH on RAD (Paired t-test, n pairs=7, *P* = 0.0656), FoG (Paired t-test, n pairs=7, *P* = 0.0138), MIC (Paired ttest, n pairs=7, *P* = 0.2308), and SMG (Paired t-test, n pairs=7, *P* = 0.0603). (c,d) DDAs (c) and BMDAs (d) measuring the effect of temperature on RAD (One way ANOVA, n groups=3, *P* = 0.6382), FoG (One way ANOVA, n groups=3, *P* = 0.0302), MIC (One way ANOVA, n groups=4, *P* = 0.9646), and SMG (One way ANOVA, n groups=4, *P* = 0.7366). (e)

DDAs examining the effect of media conditions on RAD (One way ANOVA, n groups=3, $P = 0.68$) and FoG (One way ANOVA, n groups=3, $P = 0.81$). (f) DDAs performed on casitone and YPD for a series of SC5314 derived strains with different FoG levels (paired t-tests for RAD, $P < 0.001$ and FoG, $P = 0.013$). (g) BMDAs performed with SC5314 at different initial inoculum sizes. MIC (One way ANOVA, n groups=5, $P = 1$), SMG (One way ANOVA, n groups=5, $P = 0.87$). For panels (a), (c), and (e), images of DDAs are shown for strain SC5314. RAD/MIC and FoG/SMG levels were calculated for range the of *C. albicans* strains as in Fig. 1. For all panels, $n \geq 2$; ***, $P < 0.001$; **, $P < 0.01$; *, $P < 0.05$, error bars denote standard deviations.

**Supplementary Figure 4**

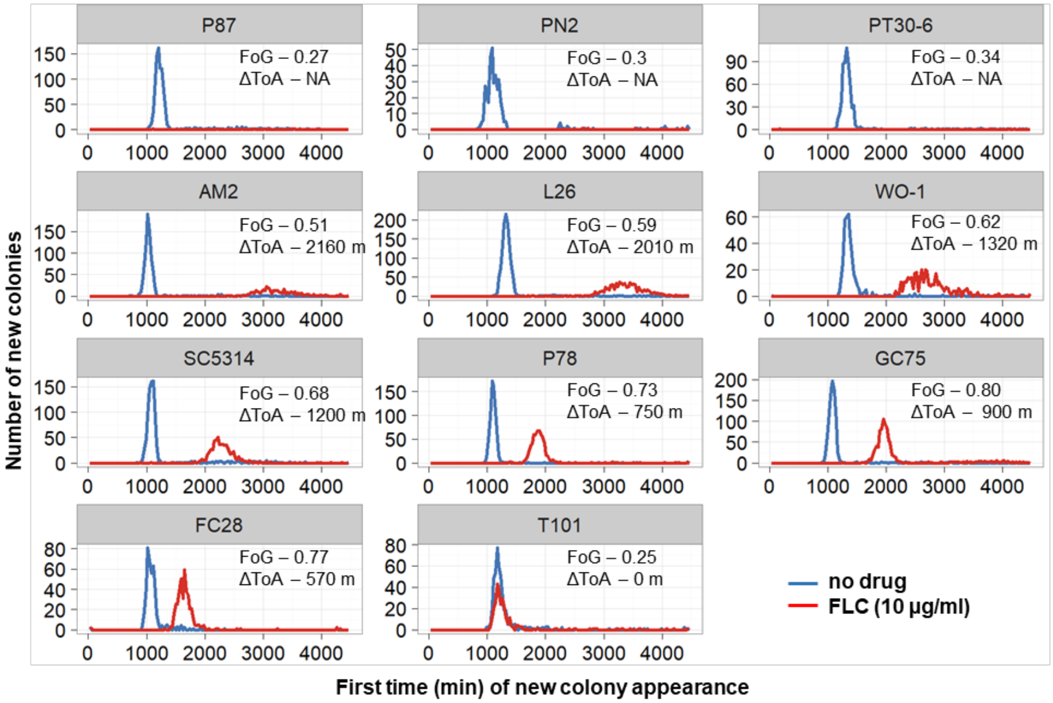


**Supplementary Figure 4.** Time of initial colony appearance (ToA) for strains with a range of FoG levels. ToA, determined using ScanLag, on a range of isolates with different FoG levels. ΔToA indicates the difference in the time of appearance of colonies with vs without drug (ΔToA = ToA with 10 µg/ml FLC – ToA without FLC). All isolates were tested with two or more biological replicates ($n \geq 2$).

## Supplementary Figure 5

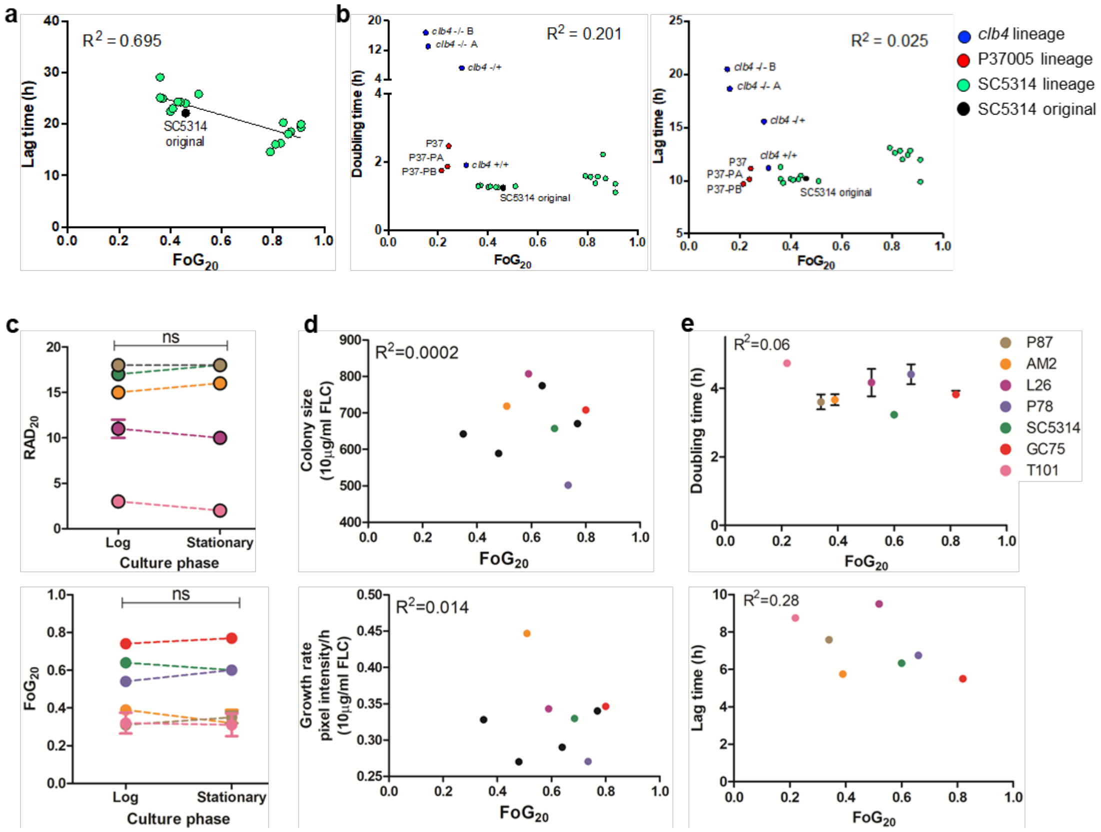


**Supplementary Figure 5.** Relationships between growth parameters and FoG. (a-b) Growth parameters (lag and doubling times) for growth in liquid were determined using BioTek Gen5 software. Data points represent the average of 3 biological replicates. (a) Correlation analysis between FoG and lag time during growth in YPD supplemented with FLC (1 µg/ml) for a series of SC5314 derived isolates with different FoG levels. (b) Correlation analyses between FoG and doubling time (left panel) or FoG and lag time (right panel) in liquid YPD without drug for 3 *C. albicans* lineages, including the P37055 lineage (P37 parental strain, and P37-PA,B passaged isolates[32], red), the *clb4* lineage including mutants of the mitotic cyclin *CLB4*[33] (BWP17 *clb +/+*, *clb +/-*, *clb -/-* A,B6, blue), and the SC5314 derived lineage (isolates passaged in YPD or YPD + FLC for 12 days, green). (c) DDAs performed on log phase and exponentially

growing cells. RAD (Paired t-test, t=0, df=6, n pairs=7, $P = 1$), FoG (Paired t-test, t=0.42, df=6, n pairs=7, $P = 0.68$). (d) Correlation analyses for FoG and colony size (upper panel) or FoG and growth rate (lower panel) on casitone plates with 10 µg/ml FLC (n = 9). (e) Correlation analyses between FoG and doubling time (upper panel) or FoG and lag time (lower panel) for *C. albicans* isolates (n = 7, $P = 0.57$ for FoG vs doubling time, $P = 0.22$ for FoG vs lag time) in liquid YPD without FLC. For all panels, $n \geq 2$; ns, $P > 0.05$, error bars denote standard deviations.

**Supplementary Figure 6**

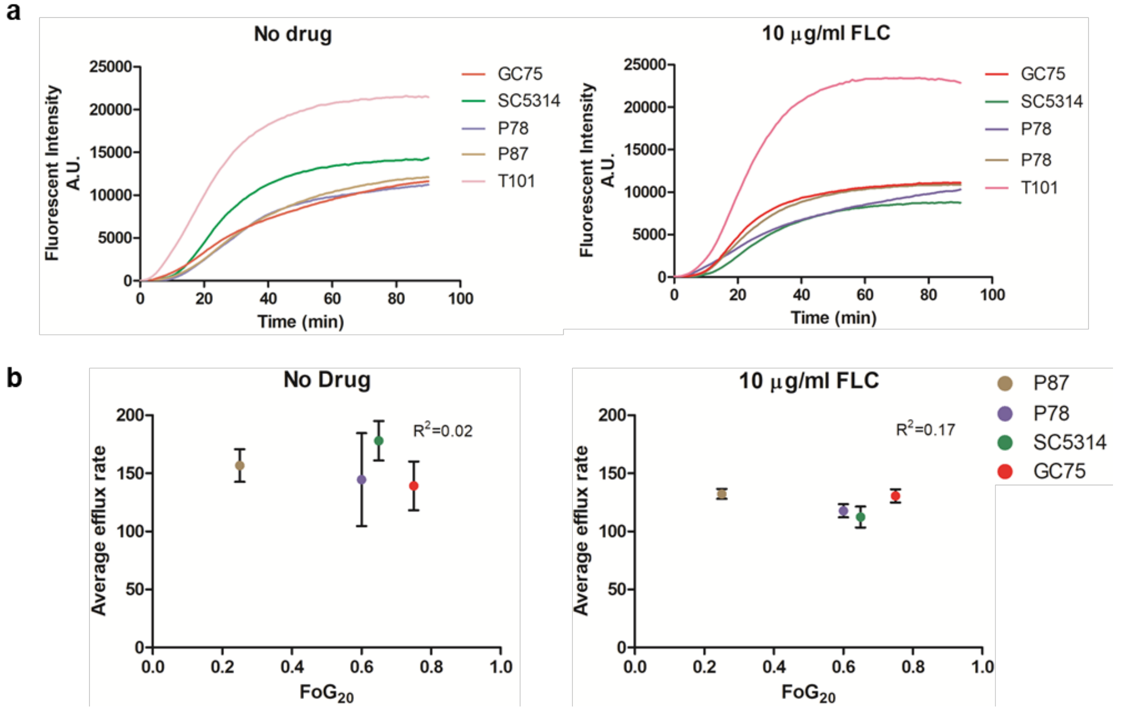


**Supplementary Figure 6.** Drug uptake and efflux of cells grown in FLC. (a) Measurement of efflux of Rhodamine 6G (R6G). Fluorescence was recorded at 1 min intervals in triplicate experiments for 90 min. Efflux curves represent one of two biological repeats shown as ΔFluorescent intensity (with Glucose - without Glucose) without drug (left) and with 10 µg/ml FLC (right). (b) Correlation analysis between efflux activity and FoG. Efflux rate of R6G in the absence of drug (left) Efflux rate of R6G in 10 µg/ml FLC (right). For all panels, $n \geq 2$; error bars denote standard deviations.

## Supplementary Figure 7

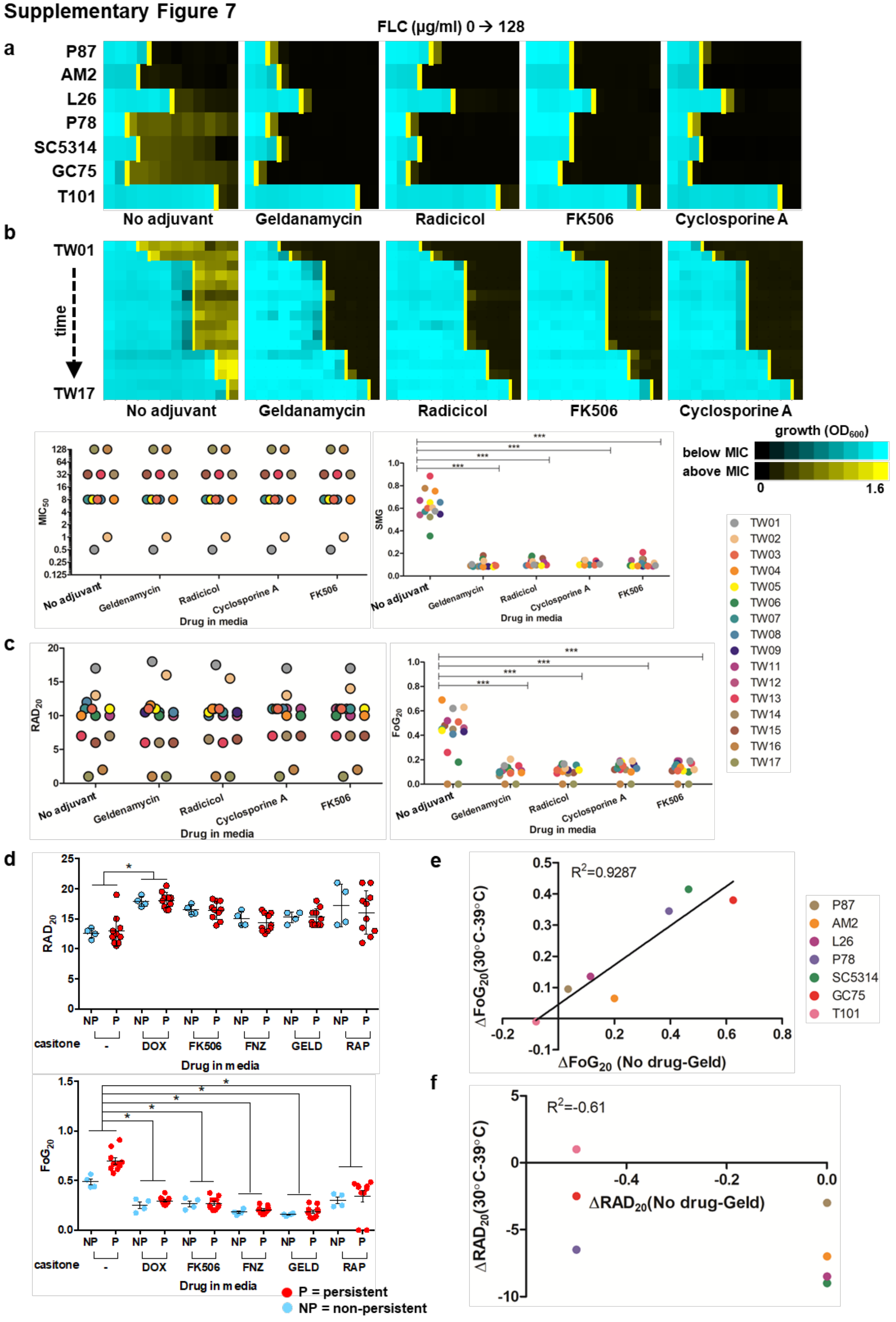

**Supplementary Figure 7, continued**

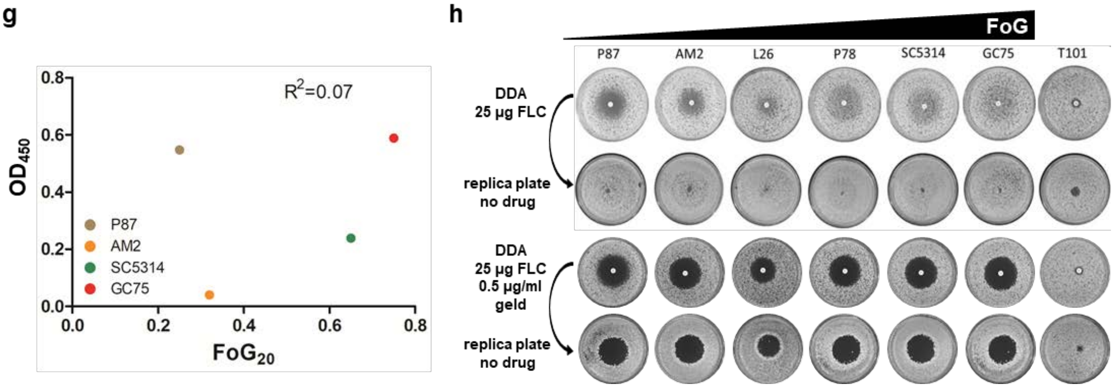


**Supplementary Figure 7.** FLC together with adjuvants significantly reduce FoG/SMG. (a,b) BMDAs on casitone supplemented with 0.5 µg/ml geldanamycin, 0.5 µg/ml radicicol, 0.5 µg/ml FK506 or 0.4 µg/ml cyclosporine A for 7 isolates (a) and 16 clinical isolate series[34] from an HIV patient. (b). $MIC_{50}$ and SMG values were coloured and calculated as in Fig. 1E. Graphs show MIC (One-way ANOVA, n groups=5, P = 1) and SMG (One-way ANOVA, n groups=5, $P < 0.0001$) levels for the HIV series. (c) RAD and FoG levels from FLC DDAs performed on casitone media supplemented with Hsp90 inhibitors (0.5 µg/ml geldanamycin or 0.5 µg/ml radicicol) or calcineurin inhibitors (0.5 µg/ml FK506 or 0.4 µg/ml cyclosporine A) on the series of 16 clinical isolates and analysed using *diskImageR.* RAD (One-way ANOVA, n groups=5, P = 0.9999) and FoG (One-way ANOVA, n groups=5, $P < 0.0001$) levels. (d) FLC DDAs for a subset of non-persistent (NP, n = 4, cyan) and persistent (P, n = 10, red) isolates from Fig. 6 measured at 48 h on casitone media supplemented with adjuvant drugs (doxycycline 50 µg/ml, 0.5 µg/ml FK506, 10 µg/ml fluphenazine, 0.5 µg/ml geldanamycin or 0.5 ng/ml rapamycin) (for RAD, $P < 0.001$ for no drug vs DOX, $P > 0.05$ for the other drugs; for FoG, $P < 0.01$ for all drug comparisons). (e, f) Correlation analyses for $\Delta FoG_{20}$ (e) and $\Delta RAD_{20}$ (f) measured in different growth conditions (casitone with no drug - casitone with 0.5 µg/ml Geldanamycin) vs (casitone at 30°C -

casitone at 39°C, n=7).  (g) Correlation analysis between Hsp90 protein levels, measured by ELISA, after 6 h of FLC induction vs FoG (n=4, $P = 0.74$).  (h) Effect of geldanamycin on viability of cells growing inside the zone of inhibition.  FLC DDAs on casitone (1st row) or casitone supplemented with 0.5 µg/ml geldanamycin (3rd row), were replica-plated onto casitone without drug and incubated at 30°C for 48 h ($2^{nd}$ and $4^{th}$ rows).  For all panels, $n \geq 2$; ***, $P < 0.001$; **, $P < 0.01$; *, $P < 0.05$, error bars denote standard deviations.

Supplementary Figure 8

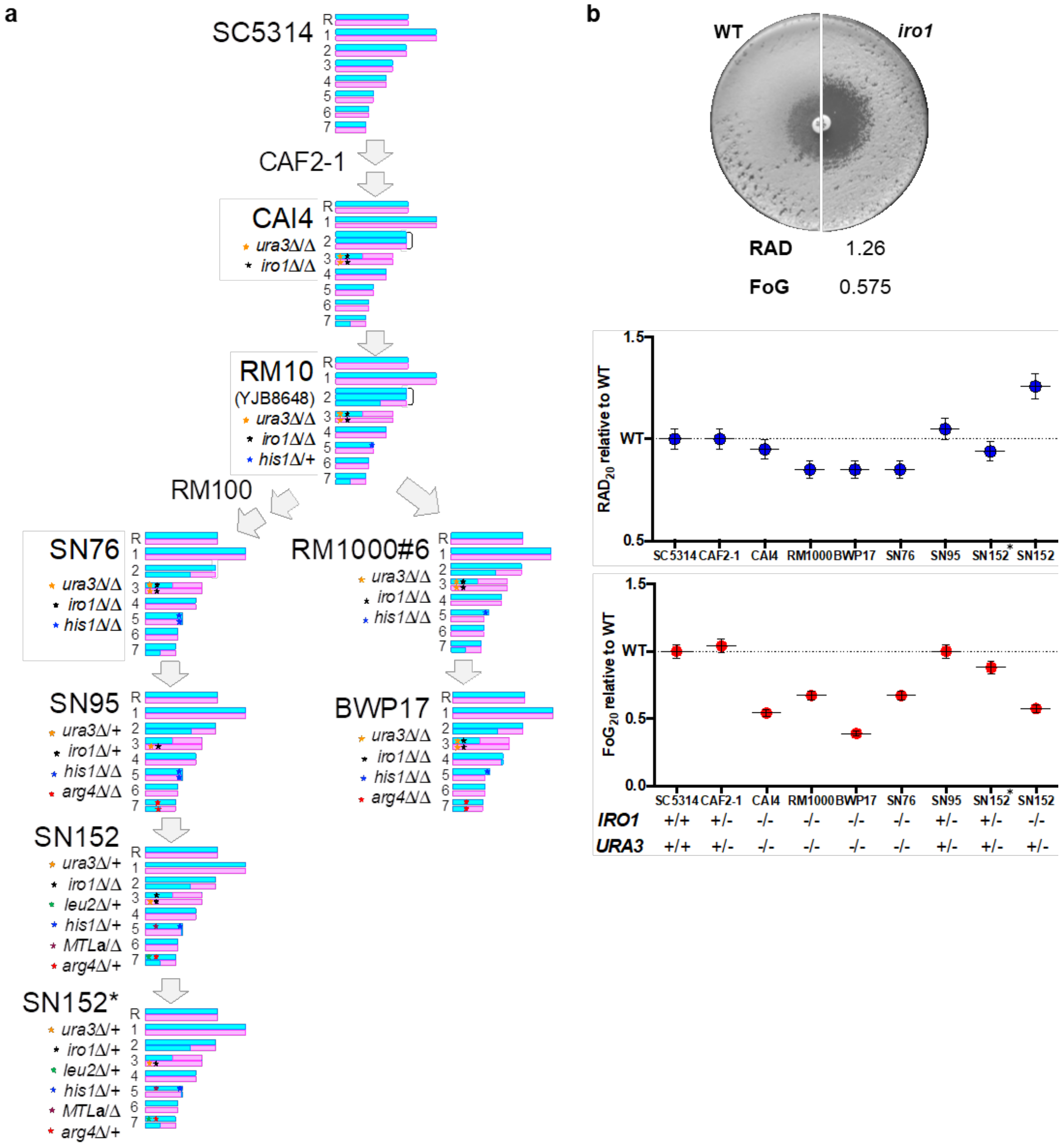


**Supplementary Figure 8**. Impact of *IRO1* on FoG levels in a standard laboratory lineage. SC5314 lineage and its derivatives CAF2-1, CAI4, RM10, RM1000, BWP17, SN76, SN95, and SN152 strain contain one or no *IRO1* alleles present. (a) Cartoon of strain haplotypes: cyan indicates allele A and magenta indicates allele B; stars indicate location of deleted genes on the respective chromosomes. (b) FLC DDAs performed using a series of laboratory strains in which the *IRO1* gene was inadvertent deleted or

reintegrated. +/- signs indicate presence or absence of *IRO1* (+/+, both *IRO1* alleles present, +/-, one *IRO1* allele present, -/- no *IRO1* alleles present). RAD and FoG levels are shown relative to the most ancestral isolate in the lineage (SC5314). Pictures show FLC DDAs performed on an *iro1* null mutant and corresponding parental strain. Graphs show RAD (top) and FoG (bottom) for strains SC5314, CAF2-1, CAI4, RM1000, BWP17, SN76, SN95, and 2 versions of the SN152 strain containing one or no *IRO1* alleles present. For panel (b), $n \geq 2$ and error bars denote standard deviations.

## Supplementary Figure 9

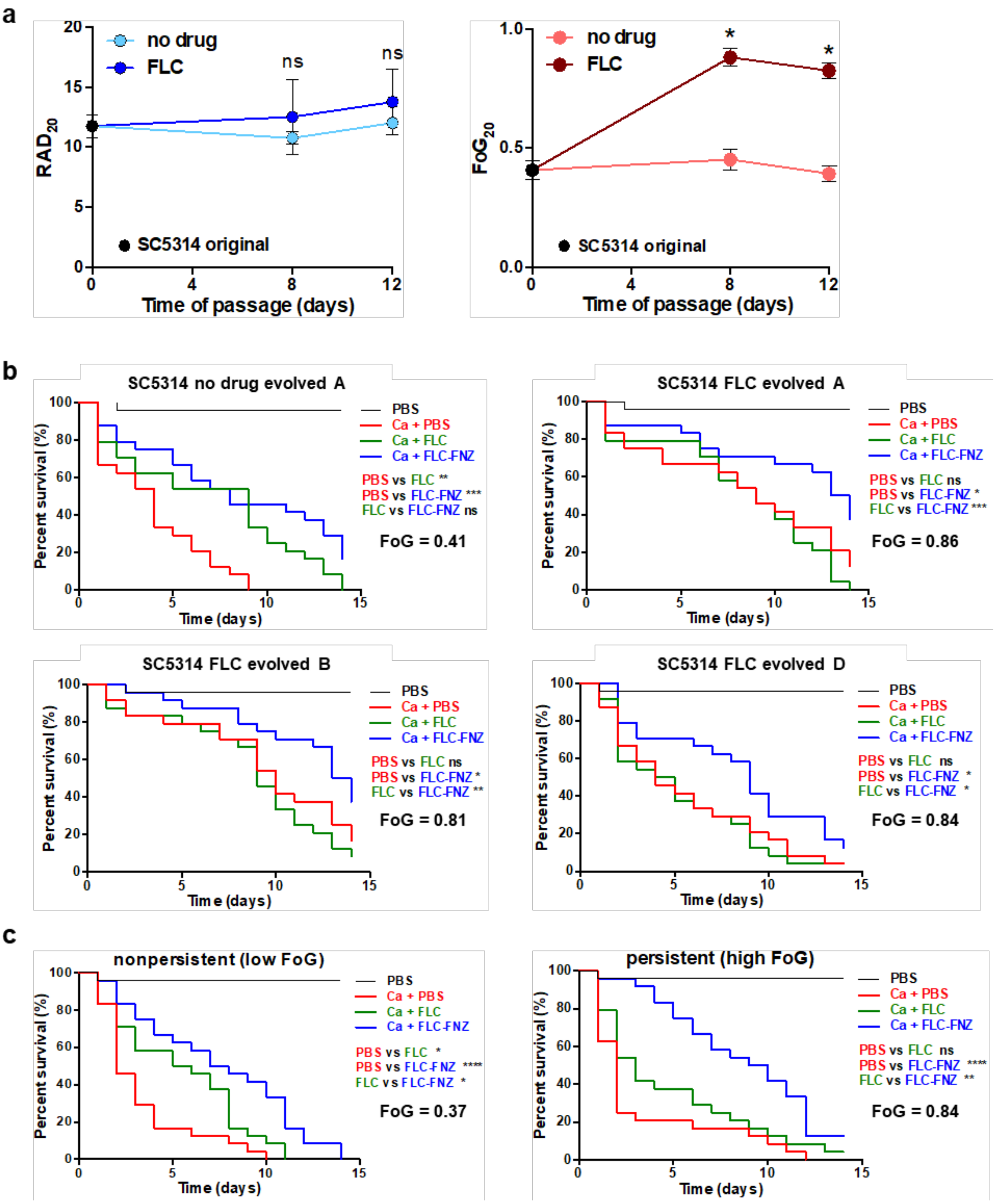


**Supplementary Figure 9**. Combination therapy partially rescues systemic infection of *G. mellonella* by high FoG *C. albicans* isolates. (a) FLC DDAs performed on a series of SC5314 lab-derived isolates. Isolates were passaged for 12 days in YPD with or without FLC. Isolates were tested in 3 or more biological replicates ($n \geq 3$), ns, $P >$

0.05, *, $P < 0.05$, and error bars denote standard deviations. (b) Survival curves of *G. mellonella* larvae infected with the SC5314 low FoG and high FoG isogenic derivatives. (c) Survival curves of *G. mellonella* larvae infected with example persistent and non-persistent strains. For (b-c), each curve represents a group of 24 larvae ($n = 24$) which were monitored daily for survival 14 days after *C. albicans* infection. *P* values represent results of log-rank test comparing different treatment conditions with significance values as follows: ns, $P > 0.05$; *, $P < 0.05$; **, $P < 0.01$; ***, $P < 0.001$.

**Supplementary Figure 10**

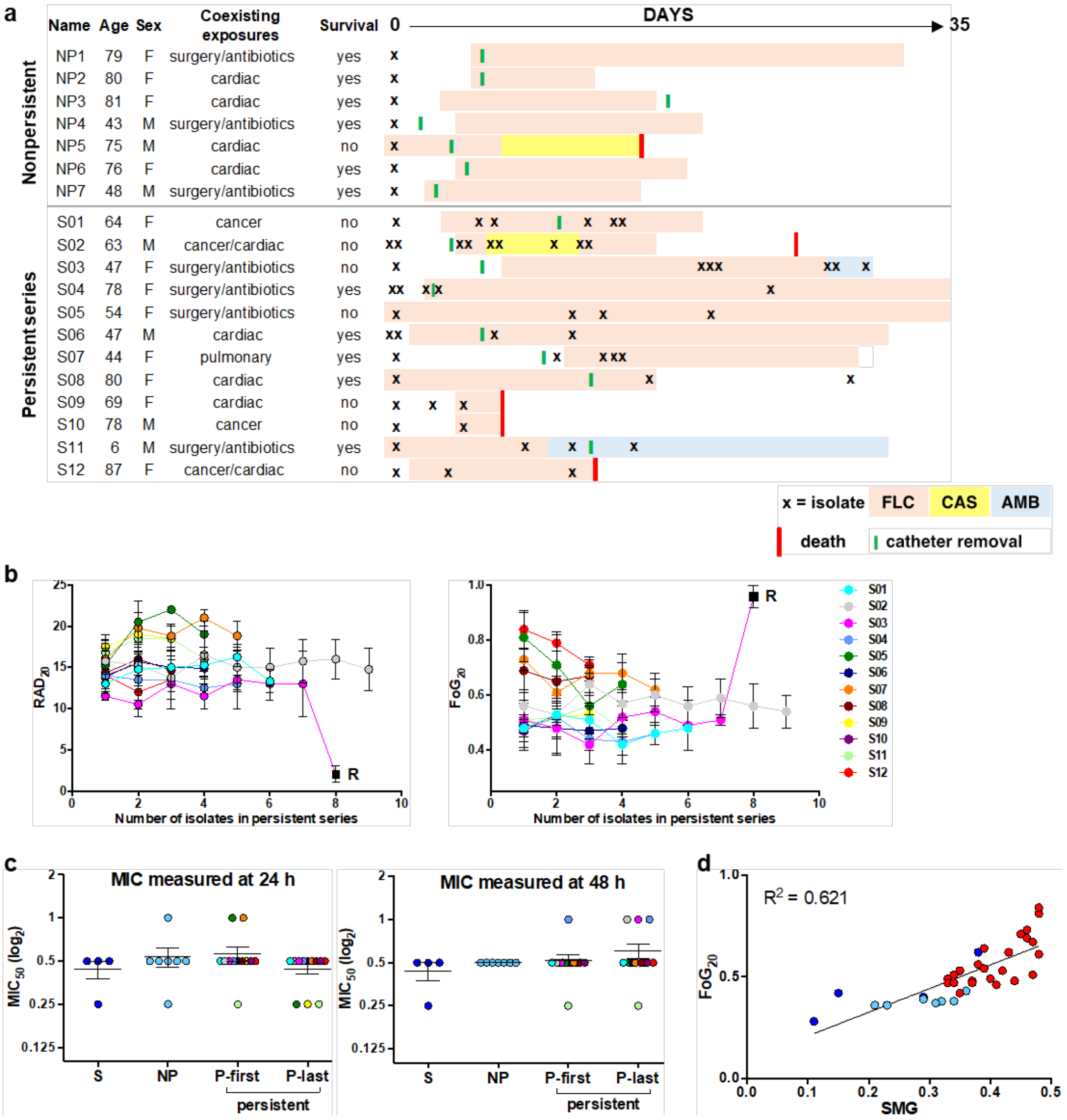


**Supplementary Figure 10**. Persistent and non-persistent strains differ significantly in tolerance measured as FoG or SMG. (a) Clinical history of 7 non-persistent isolates and 12 series of clinically persistent isolates showing the age, sex and associated conditions of the patients from whom they were isolated. ‘X’ symbols indicate the time when isolates were collected, shaded areas indicate the duration of antifungal therapy received (FLC, pink; caspofungin, yellow; amphotericin B, blue), red lines mark death events over a 30-day follow-up. (b) FLC DDAs performed on all isolates from each

patient series with a *Candida* persistent infection (see Fig. 6A for isolates) were analysed using *diskImageR* for RAD (left) and FoG (right). The final isolate in the S03 series became resistant during FLC therapy and is marked in black (R). (c) MIC levels from BMDAs at 24 h and 48 h for drug-susceptible isolates (SC5314, GC75, P78042 and P87), non-persistent isolates (NP1-7), and the first and the last isolate from each of the persistent series (S01-12). The final isolate in S03 became FLC resistant (R), therefore the penultimate isolate in this series was included. (d) Correlation analysis between FoG and SMG levels for reference isolates (dark blue), first and last isolates in the persistent series (red) and non-persistent isolates (light blue) (n = 35, $P < 0.0001$) shows that FoG and SMG correlate well for these clinical isolates as they do for isolates analysed previously (Supplementary Fig. 2c, middle panel). For all panels, $n \geq 3$ and error bars denote standard deviations.

**Supplementary Table 1.** Percent cell viability measured after 16 h in the presence of 10 µg/ml FLC using Propidium iodide (PI) staining, $n \geq 2$, SD = standard deviations.

| **Strain** | **% cell viability** | |
|---|---|---|
| | **Average** | **SD** |
| GC75 | 97.84 | 1.20 |
| FC28 | 94.12 | 1.04 |
| P78 | 98.38 | 0.20 |
| SC5314 | 98.22 | 0.71 |
| PT30-8 | 96.40 | 0.95 |
| L26 | 96.34 | 2.28 |
| WO-1 | 98.20 | 2.28 |
| PN2 | 97.12 | 3.93 |
| AM2 | 98.14 | 0.95 |
| PT30-6 | 95.11 | 2.55 |
| P87 | 94.96 | 2.55 |
| T101 | 98.72 | 1.04 |

**Supplementary Table 2.** RAD and FoG levels on casitone medium of the SC5314-derived laboratory strain lineage, $n \geq 2$.

| **Strain** | **$RAD_{20}$** | **$FoG_{20}$** | ***URA3*** | ***IRO1*** |
|---|---|---|---|---|
| SC5314 | 1.00 | 1.00 | *ura3+/+* | *iro+/+* |
| CAF2-1 | 1.00 | 1.04 | *ura3+/-* | *iro+/-* |
| CAI4 | 0.95 | 0.54 | *ura3-/-* | *iro-/-* |
| CAI4 + *URA3* | 0.95 | 0.54 | *ura3-/-* | *iro-/-* |
| RM1000 #6 | 0.85 | 0.67 | *ura3-/-* | *iro-/-* |
| BWP17 | 0.85 | 0.39 | *ura3-/-* | *iro-/-* |
| RM1000 #2 | 0.90 | 0.70 | *ura3-/-* | *iro-/-* |
| SN76 | 0.85 | 0.67 | *ura3-/-* | *iro-/-* |
| SN95 | 1.05 | 1.00 | *ura3+/-* | *iro+/-* |
| SN152* | 1.13 | 0.94 | *ura3+/-* | *iro+/-* |
| SN152* *iro1Δ* | 1.26 | 0.57 | *ura3+/-* | *iro-/-* |

**Supplementary Table 3.** RAD and FoG levels on casitone medium of null mutants of interest, $n \geq 2$.

| Function/pathway tested | Relevant wild-type | Mutant | $RAD_{20}$ | $FoG_{20}$ |
|---|---|---|---|---|
| calcineurin pathway | SN152 | SN152 | 1.00 | 1.00 |
| | | *cnb1*ΔΔ | 1.00 | 0.48 |
| | | *crz1*ΔΔ | 1.10 | 0.39 |
| calcineurin regulators | SC5314 | *rcn1*ΔΔ *crz1*ΔΔ | 1.00 | 0.46 |
| | SN95 | *rcn1*ΔΔ | 1.05 | 0.94 |
| | | *rcn2*ΔΔ | 1.05 | 0.91 |
| vacuole/intracellular calcium concentration | BWP17 prototroph | BWP17 prototroph | 1.00 | 1.00 |
| | | *vps21*ΔΔ | 1.00 | 2.34 |
| cell wall maintenance kinase | RBY717 | *mkc1*ΔΔ | 1.21 | 0.58 |
| regulator of ergosterol biosynthesis genes | SN152 | *upc2*ΔΔ | 1.25 | 0.29 |
| regulators of efflux pump genes | | *tac1*ΔΔ | 1.05 | 0.95 |
| | | *mrr1*ΔΔ | 1.10 | 0.89 |

**Supplementary Table 4.** Plasmids and primers used in this study (BJB-T2 and BJB-T61 are described in refs. [35] and [36], respectively).

| Primer | Sequence |
|---|---|
| BP1440 | 5' CCACTCCTTTTCCTTATTTCCCAAATCAACCAATTATTCTTT TTCTTACTCTTTA TTTGCgttttcccagtcacgacgtt 3' |
| BP1441 | 5' GTGATTGTAGGGGACGATACAAAATCTTGTTCTTTTTGTTT TTCATTAAGGAGA CCAGGCtgtggaattgtgagcggata 3' |
| BP1444 | 5' gtgctctccgcgacaatatctt 3' |
| BP1445 | 5' ccacgagacacgacctaataat 3' |

| Name | Plasmid | Reference |
|---|---|---|
| BJB-T2 | pGEM-His1 | 35 |
| BJB-T61 | pSN69-Arg4 | 36 |

## Supplementary References


1. Bjerknes, R. Flow cytometric assay for combined measurement of phagocytosis and intracellular killing of *Candida albicans*. *J Immunol Methods* **72**, 229-41 (1984).
2. Garcia, M.G. *et al.* Isolation of a *Candida albicans* gene, tightly linked to *URA3*, coding for a putative transcription factor that suppresses a *Saccharomyces cerevisiae aft1* mutation. *Yeast* **18**, 301-11 (2001).
3. Hameed, S., Dhamgaye, S., Singh, A., Goswami, S.K. & Prasad, R. Calcineurin signalling and membrane lipid homeostasis regulates iron mediated multidrug resistance mechanisms in *Candida albicans*. *PLoS One* **6**, e18684 (2011).
4. Bensen, E.S., Martin, S.J., Li, M., Berman, J. & Davis, D.A. Transcriptional profiling in *Candida albicans* reveals new adaptive responses to extracellular pH and functions for Rim101p. *Mol Microbiol* **54**, 1335-51 (2004).
5. Garnaud, C. *et al.* The Rim pathway mediates antifungal tolerance in *Candida albicans* through newly identified Rim101 transcriptional targets including Hsp90 and Ipt1. *Antimicrob Agents Chemother* (2018). pii: e01785-1. doi: 10.1128/AAC.01785-17.
6. Vasicek, E.M. *et al.* Disruption of the transcriptional regulator Cas5 results in enhanced killing of *Candida albicans* by Fluconazole. *Antimicrob Agents Chemother* **58**, 6807-18 (2014).
7. Liu, S. *et al.* Synergistic Effect of fluconazole and calcium channel blockers against resistant *Candida albicans*. *PLoS One* **11**, e0150859 (2016).

8. Marchetti, O., Moreillon, P., Glauser, M.P., Bille, J. & Sanglard, D. Potent synergism of the combination of fluconazole and cyclosporine in *Candida albicans*. *Antimicrob Agents Chemother* **44**, 2373-81 (2000).
9. Uppuluri, P., Nett, J., Heitman, J. & Andes, D. Synergistic effect of calcineurin inhibitors and fluconazole against *Candida albicans* biofilms. *Antimicrob Agents Chemother* **52**, 1127-32 (2008).
10. Fiori, A. & Van Dijck, P. Potent synergistic effect of doxycycline with fluconazole against *Candida albicans* is mediated by interference with iron homeostasis. *Antimicrob Agents Chemother* **56**, 3785-96 (2012).
11. Onyewu, C., Blankenship, J.R., Del Poeta, M. & Heitman, J. Ergosterol biosynthesis inhibitors become fungicidal when combined with calcineurin inhibitors against *Candida albicans*, *Candida glabrata*, and *Candida krusei*. *Antimicrob Agents Chemother* **47**, 95664 (2003).
12. Epp, E. *et al.* Reverse genetics in *Candida albicans* predicts ARF cycling is essential for drug resistance and virulence. *PLoS Pathog* **6**, e1000753 (2010).
13. Cardenas, M.E., Sanfridson, A., Cutler, N.S. & Heitman, J. Signal-transduction cascades as targets for therapeutic intervention by natural products. *Trends Biotechnol* **16**, 427-33 (1998).
14. Cruz, M.C. *et al.* Calcineurin is essential for survival during membrane stress in *Candida albicans*. *EMBO J* **21**, 546-59 (2002).
15. Onyewu, C., Wormley, F.L., Jr., Perfect, J.R. & Heitman, J. The calcineurin target, Crz1, functions in azole tolerance but is not required for virulence of *Candida albicans*. *Infection and immunity* **72**, 7330-7333 (2004).

16. Reedy, J.L., Filler, S.G. & Heitman, J. Elucidating the *Candida albicans* calcineurin signalling cascade controlling stress response and virulence. *Fungal Genet Biol* **47**, 10716 (2010).

17. Miyazaki, T. *et al.* Functional characterization of the regulators of calcineurin in *Candida glabrata*. *FEMS Yeast Res* **11**, 621-30 (2011).

18. LaFayette, S.L. *et al.* PKC signalling regulates drug resistance of the fungal pathogen *Candida albicans* via circuitry comprised of Mkc1, calcineurin, and Hsp90. *PLoS Pathog* **6**, e1001069 (2010).

19. Robbins, N., Caplan, T. & Cowen, L.E. Molecular Evolution of Antifungal Drug Resistance. *Annu Rev Microbiol* **71**, 753-775 (2017).

20. Johnston, D.A., Eberle, K.E., Sturtevant, J.E. & Palmer, G.E. Role for endosomal and vacuolar GTPases in *Candida albicans* pathogenesis. *Infect Immun* **77**, 2343-55 (2009).

21. Luna-Tapia, A., Kerns, M.E., Eberle, K.E., Jursic, B.S. & Palmer, G.E. Trafficking through the late endosome significantly impacts *Candida albicans* tolerance of the azole antifungals. *Antimicrob Agents Chemother* **59**, 2410-20 (2015).

22. Vasicek, E.M., Berkow, E.L., Flowers, S.A., Barker, K.S. & Rogers, P.D. *UPC2* is universally essential for azole antifungal resistance in *Candida albicans*. *Eukaryot Cell* **13**, 933-46 (2014).

23. Sasse, C. *et al.* The transcription factor Ndt80 does not contribute to Mrr1-, Tac1-, and Upc2-mediated fluconazole resistance in *Candida albicans*. *PLoS One* **6**, e25623 (2011).

24. Schubert, S. *et al.* Regulation of efflux pump expression and drug resistance by the transcription factors Mrr1, Upc2, and Cap1 in *Candida albicans*. *Antimicrob Agents Chemother* **55**, 2212-23 (2011).

25. Homann, O.R., Dea, J., Noble, S.M. & Johnson, A.D. A phenotypic profile of the *Candida albicans* regulatory network. *PLoS genetics* **5**, e1000783 (2009).

26. Pfaller, M.A. Antifungal drug resistance: mechanisms, epidemiology, and consequences for treatment. *Am J Med* **125**, S3-13 (2012).

27. Prasad, R., Gaur, N.A., Gaur, M. & Komath, S.S. Efflux pumps in drug resistance of *Candida*. *Infect Disord Drug Targets* **6**, 69-83 (2006).

28. Hoot, S.J., Brown, R.P., Oliver, B.G. & White, T.C. The UPC2 promoter in *Candida albicans* contains two cis-acting elements that bind directly to Upc2p, resulting in transcriptional autoregulation. *Eukaryot Cell* **9**, 1354-62 (2010).

29. Wellington, M., Koselny, K., Sutterwala, F.S. & Krysan, D.J. *Candida albicans* triggers NLRP3-mediated pyroptosis in macrophages. *Eukaryot Cell* **13**, 329-40 (2014).

30. Park, Y.N. & Morschhauser, J. Tetracycline-inducible gene expression and gene deletion in *Candida albicans*. *Eukaryot Cell* **4**, 1328-42 (2005).

31. Weyler, M. & Morschhauser, J. Tetracycline-inducible gene expression in *Candida albicans*. *Methods Mol Biol* **845**, 201-10 (2012).

32. Alby, K. & Bennett, R.J. Stress-induced phenotypic switching in *Candida albicans*. *Molecular biology of the cell* **20**, 3178-3191 (2009).

33. Bensen, E.S., Clemente-Blanco, A., Finley, K.R., Correa-Bordes, J. & Berman, J. The mitotic cyclins Clb2p and Clb4p affect morphogenesis in *Candida albicans*. *Mol Biol Cell* **16**, 3387-400 (2005).

34. White, T.C. Increased mRNA levels of *ERG16*, *CDR*, and *MDR1* correlate with increases in azole resistance in *Candida albicans* isolates from a patient infected with human immunodeficiency virus. *Antimicrob Agents Chemother* **41**, 1482-7 (1997).

35. Wilson, R.B., Davis, D. & Mitchell, A.P. Rapid hypothesis testing with *Candida albicans* through gene disruption with short homology regions. *Journal of Bacteriology* **181**, 1868-1874 (1999).

36. Noble, S.M. & Johnson, A.D. Strains and strategies for large-scale gene deletion studies of the diploid human fungal pathogen *Candida albican*s. *Eukaryotic cell* **4**, 298-309 (2005).
