# Supplementary material for: Antifungal tolerance is a subpopulation effect distinct from resistance and is associated with persistent candidemia
Source: Nat Commun. 2018 Jun 25;9:2470. doi: 10.1038/s41467-018-04926-x (PMC6018213; doi:10.1038/s41467-018-04926-x)
Supplement: Supplementary file 2 — Description of Additional Supplementary Files [file 41467_2018_4926_MOESM2_ESM.pdf]

Description of Additional Supplementary Files

File Name: Supplementary Data 1
Description: Strains used in this study.

File Name: Supplementary Data 1
Description: Detailed clinical information on persistent and nonpersistent isolates, including MIC levels, antifungal therapy, catheter information and coexisting exposures.

File Name: Supplementary Movie 1
Description: Time-lapse video of P87 cells in FLC. This strain has low FoG (FoG=0.29, RAD=18). Cells were plated in liquid casitone medium containing 10 µg/ml FLC, which is ~20-fold above their MIC levels.

File Name: Supplementary Movie 2
Description: Time-lapse video of AM2 cells in FLC. This strain has medium FoG (FoG=0.41, RAD=17). Cells were plated in liquid casitone medium containing 10 µg/ml FLC, which is ~20-fold above their MIC levels.

File Name: Supplementary Movie 3
Description: Time-lapse video of P78 cells in FLC. This strain has medium-high FoG (FoG=0.62, RAD=18.5). Cells were plated in liquid casitone medium containing 10 µg/ml FLC, which is ~20-fold above their MIC levels.

File Name: Supplementary Movie 4
Description: Time-lapse video of SC5314 cells in FLC. This strain has high FoG (FoG=0.68, RAD=18). Cells were plated in liquid casitone medium containing 10 µg/ml FLC, which is ~20-fold above their MIC levels.
